# “Keeping the Light On”: A Qualitative Study on Hope Perceptions at the End of Life in Portuguese Family Dyads

**DOI:** 10.3390/ijerph19031561

**Published:** 2022-01-29

**Authors:** Carlos Laranjeira, Maria Anjos Dixe, Isabel Semeão, Sara Rijo, Catarina Faria, Ana Querido

**Affiliations:** 1 School of Health Sciences, Polytechnic of Leiria, Campus 2, Morro do Lena, Alto do Vieiro, Apartado 4137, 2411-901 Leiria, Portugal; maria.dixe@ipleiria.pt (M.d.A.D.); ana.querido@ipleiria.pt (A.Q.); 2 Centre for Innovative Care and Health Technology (ciTechCare), Rua de Santo André—66-68, Campus 5, Polytechnic of Leiria, 2410-541 Leiria, Portugal; 3 Research in Education and Community Intervention (RECI I&D), Piaget Institute, 3515-776 Viseu, Portugal; 4 Hospital Palliative Care Team, Hospital Center of Leiria-Hospital de Santo André, R. de Santo André, 2410-197 Leiria, Portugal; isabel.tomar@gmail.com (I.S.); sara.f.rijo@gmail.com (S.R.); catarina.faria@chleiria.min-saude.pt (C.F.); 5 Palliative Care Unit, Hospital Center of Leiria-Hospital Bernardino Lopes de Oliveira, Rua do Hospital–Apartado 70, 2460-051 Alcobaça, Portugal; 6 Center for Health Technology and Services Research (CINTESIS), NursID, University of Porto, 4200-450 Porto, Portugal

**Keywords:** hope, palliative care, end-of-life, family dyads, Portugal

## Abstract

Hope performs an important role in how patients and their families cope with suffering and stressful events. To better inform practice and theory on hope, palliative care research should include both patients and their family carers, given their strong interdependence. The aim of this study was to explore how hope is experienced in dyads formed by end-of-life patients and their family carers. In this qualitative study, data were collected by in-depth interviews with seven Portuguese family dyads. Analysis followed a thematic analysis approach. The analysis of the interviews shed light on the importance of hope for all participants, and the challenges involved. Family dyads noted several barriers and facilitators to perceptions of hope. Barriers to hope included limitations imposed by illness, feelings of anguish and helplessness, and poor communication with clinicians. Hope facilitators included supportive others, positive thinking and sense of humour, connection with nature, faith in religion and science, and a sense of compassion with others and altruism. Given the multidimensional scope of hope, the main challenge for family dyads is to look beyond the disease itself. Thus, palliative care teams should be encouraged to support and foster realistic hope, helping families prepare for death, in the context of advanced cancer.

## 1. Introduction

In palliative care, the way patients balance feelings of hope and hopelessness can play a significant role in how they perceive their quality of life and quality of dying [1,2,3]. Hope is central to an individual’s existence and can be understood to have both an external, objective dimension (i.e., out there somewhere) and an internal, subjective dimension (i.e., pertaining to the individual). Hope is also an action with several dimensions, including cognitive, behavioural, and affiliative, which are influenced by an individual’s context and perspective of time [1]. More than any other mental state, hope is a balance between cognition and emotion, strongly oriented towards the future and the expectation of positive outcomes [4,5].

Previous studies suggest that sustaining hope can have a positive impact on patient outcomes, such as quality of life [6,7,8,9]. In addition, hope can be an important facilitator to cope with difficult situations and is associated with finding meaning through inner resources [10,11,12,13,14]. A recent systematic review [15] found that psychotherapy is the most common intervention used to increase hope, and may also improve spiritual well-being and reduce depression. Similarly, dignity therapy, as an individualised psychotherapy, can be offered as an effective intervention for terminally ill patients to reduce their anxiety and depression [16].

Hope plays a key role in providing comfort and quality of life for patients and families [17] and was found to predict resilience [18]. In contrast, hopelessness is associated with anxiety, spiritual suffering, depression, suicidal ideation, and physical illness [13,17]. Hope is predominantly viewed as something positive that should be nurtured and fostered by healthcare providers in their daily interactions with patients and families; although, clinicians may view hope negatively if it is disconnected from medical reality, especially in palliative care. There is concern that discussing bad news, such as communicating a diagnosis of incurable cancer or planning for end-of-life (EOL), may decrease hope. This is one reason such conversations are avoided [19]. There is agreement, however, that clear, honest communication is both desirable and necessary to foster a trusting relationship among patient, family, and healthcare professional, as well as planning for the future [1,20,21]. Therefore, the aim of supporting hope may conflict with that of communicating honestly, particularly about difficult topics such as EOL issues. This can negatively influence clinical practice, for example, by delaying referral to palliative care [22].

Researchers admit that unrealistic hope—such as hope for a miracle, divine intervention, or a new miraculous treatment—has a unique status for patients in EOL care, who can harbour unrealistic forms of hope [23,24]. Despite some theoretical discussion, few empirical studies have explored how “false” or “unrealistic” hope can influence patients and their families. In addition, the experience and need for hope in dyads of terminally ill adult patients and their family carers has received scant attention. In Portugal, so far, there has been no study of hope perceptions based on carer–patient dyads at EOL. Portuguese culture is deeply embedded in the Catholic religion; for that reason, death and dying are still taboo topics [25]. A fatalistic belief, characterized by persistent melancholy and pessimism, is a relevant attribute of the Portuguese collective psyche [25].

This study focused primarily on exploring how hope is experienced by dyads of EOL patients and their family carers, and identifying factors that constrain or facilitate a dyad’s shared perception of hope and how it is provided and received. This empirical information is critical for recommendations on how to develop hope-based interventions for family dyads.

## 2. Materials and Methods

### 2.1. Study Design and Research Paradigm

An exploratory qualitative study was performed using thematic analysis within a critical realist paradigm. Critical realism views reality as complex and recognises that understanding this complexity must go beyond describing surface-level empirical observations. In fact, deeper levels of understanding and knowledge, gained through adapting methods of exploration, confer critical realism’s usefulness and philosophical fortitude [26,27]. This framework offers an epistemology and ontology for conceptualizing reality and developing theory and methodology in the natural and human sciences, but also in nursing [28].

The study is reported following the recommendation of the Consolidated Criteria for Reporting Qualitative Research (COREQ) [29].

### 2.2. Setting and Participant Recruitment

Participants were recruited in the palliative and/or hospice care unit of a Portuguese hospital, between March and May 2021. Purposive sampling was used to select participants among all known patients, and their family caregivers (including parents, spouses, relatives, or friends). The inclusion criteria of participants were as follows: (1) adult individuals (aged ≥ 18 years); (2) adequate Portuguese language skills; (3) cognitive ability to participate in the study. Patients chose an informal family caregiver (FC) to participate in the interviews. All patients were in the EOL stage of an advanced oncological illness (as determined by the physician of the palliative care team; generally considered to be the last months of life).

Individuals were included regardless of gender, age, marital situation, cultural background, and primary diagnosis. This option is aligned with critical realism and the existence of different layers of reality [27,28]. Eligibility was assessed by a recruitment partner and members of the research team. Patients with severe uncontrolled symptoms were excluded. Ten eligible family dyads were invited to participate, but one considered itself too emotionally unstable to participate and two experienced severe physical deterioration, leaving seven participating dyads in the study.

### 2.3. Data Collection

A semi-structured interview was developed by our team, using open-ended questions aimed to explore patient and FC views and experiences of hope. The interviews were guided by a topic list informed by clinical experience and the literature [5,30,31,32]. Moreover, a brief questionnaire was used to elicit socio-demographic details and to assess the symptoms of patients receiving palliative care (Portuguese version of the Edmonton Symptom Assessment Scale—ESAS) [33]. The ESAS, originally developed by Bruera et al. [34], allows for rapid assessment and interpretation of 10 symptoms: pain, activity, nausea, depression, anxiety, drowsiness, appetite, well-being, shortness of breath, and another symptom the patient might refer to [33]. The patient scores their intensity of symptom from 0 (none) to 10 (severe).

The interview guide included both broad and probing questions. Interviews started with an open question about hope in palliative care. The interviewer did not define hope nor related themes prior to the interview, to best explore the participant’s experiences and definitions. The next questions sought to identify the interviewee’s perceptions of hope in EOL care, including perceived barriers and facilitators related to hope, care management, and the relationship between physicians, staff, and family members. Participants were asked to describe whether they experienced hope, and if so, to elaborate on the content, value, and meaning of hope, and how it has changed during the illness process. Participants not reporting hope were invited to elucidate their experience. Though the guide was not pilot tested, questions were rephrased during the interviews to improve clarity and understanding.

All interviews were conducted by a nurse involved in care for EOL patients (SR). However, the nurse wore regular clothes during interviews (not a uniform), signalling that the interview was not part of, and would not affect, their therapeutic trajectory. In addition, the nurse was not directly involved in the daily care of the dyads interviewed. Face-to-face interviews were conducted simultaneously with the patient and their FCs. There were no repeat interviews. Each interview lasted between 30 and 40 min and was digitally audio-recorded for verbatim transcription by the first author shortly afterwards. The interviews took place in a private location at the local palliative unit. Notes were taken during and after the interviews. The interviews were conducted in participant’s native language. The first author translated the textual extracts presented in this article into English.

### 2.4. Data Analysis and Rigour

The analytic technique used in this research was thematic analysis [35,36]. WebQDA software (Universidade de Aveiro, Aveiro, Portugal) was used to organize and store the emergent findings [37]. To increase trustworthiness, firstly, two authors (C.L. and A.Q.) independently reviewed the transcripts and identified key themes and concepts that arose during the interviews. Second, a preliminary codebook was developed through an iterative process that began with the systematic comparison of the investigators’ respective codes and ended when the two authors reached a consensus [38]. Discrepancies were resolved by discussion with a third researcher (M.D.). Third, the transcripts were recoded using this codebook and, after final consolidation, a single researcher (C.L.) proceeded with coding the remaining interviews. Finally, themes and subthemes were defined and named, and relevant quotes were selected to document and illustrate each subtheme. Analysis ceased when no new information emerged from the interviews (data saturation) [39]. Demographics of the sample and a detailed description of the setting were provided to enable readers to judge the transferability of our results. To establish confirmability, we linked interpretations with quotes of participants and consulted field notes.

The six team members included nurses and clinicians working in palliative care (S.R.; I.S.; C.F.) and experts and researchers in qualitative health research with a nursing and palliative care background (C.L., A.Q. and M.D.). In addition, the research team adopted a critical realism position, based on acknowledging human frailty and paying attention to how individual backgrounds and history can influence experience [27].

### 2.5. Ethical Considerations

Prior to each interview, informed consent, including consent for audio recording, was obtained in written form. Patients were allowed ask questions about the study, and could request the presence of family members. Participants were interviewed during the first encounter or, if they preferred, at a later scheduled date. They were advised that they could withdraw from the study at any time. No compensation was offered in return for their voluntary participation.

The study was reviewed and conducted in accordance with the principles expressed in the Declaration of Helsinki, and the protocol was approved by the Ethics Committee of Leiria Hospital Center (approval nº49/19). Records for analysis did not include names or any other identifying characteristics of the participants. On completion of the study, all audio files, fieldnotes, and transcripts were transferred to a password protected, secure drive, behind a firewall. Only the first author had access to the transcribed material.

## 3. Findings

### 3.1. Sample Characteristics

In total, 7 patients (3 males, 4 females) were interviewed, together with their FCs (7 spouses), they were all heterosexual couples. All participants were Catholics and had a European cultural background. The mean ages of the patients and their FCs was 49.9 ± 10.6 years (range 33–66) and 47.4 ± 11.8 years (range 37–65), respectively. All patients were diagnosed with advanced progressive cancer disease (stage 4); 4 of them suffered from metastatic gastrointestinal cancer. The patient from dyad 1 died 3 weeks after data collection. Total Edmonton Symptom Assessment Scale (ESAS) scores were low (mean = 3.4 ± 1.1; range 0–9). The most prevalent symptom was fatigue (mean = 6 ± 3, range 2–9). Table 1 provides details on all participants and dyads.

### 3.2. Interviews—Coding Findings

Analysis of the interviews shed light on the importance of hope for all participants, and the challenges involved. Family dyads noted several barriers and facilitators to understanding perceptions of hope, and two main themes and eight subthemes were identified. Common elements were sought across the narratives, case by case. Importantly, none of the dyads expressed any regret over participating, nor any concerns about their experience. Rather, many commented that they “*got a lot out of* [the research interview]” (D7). Two dyads specifically expressed their gratitude for being able to talk about the topic of hope together and share their own experiences with the nurse.

We present findings in the form of interview extracts, interspersed with our own reflections and analysis.

#### 3.2.1. Understandings of Hope

All dyads identified hope in their current plan of care, although the strength and rank of this hope in relation to other hopes did vary. Throughout the interviews, other smaller hopes were quietly raised, such further treatment if necessary. D3 shared that “*a cure is not possible or is almost impossible. Still, we cannot help but believe, this is to have hope*”.

Family dyads indicated that good pain and symptom control, fundamental aspects of palliative care, often promoted hope. Almost all participants used metaphors of combat or struggle to describe their understanding of hope and their expectations regarding hope as an outcome. Many regarded their positive expectations about therapeutic benefits, even when prognosis was grim, as indications of their unabated force, signs they would not quit or “lose hope”, and would always continue to fight.

Hope was recognized through personal short-term achievements, which gave purpose in life and a positive sense of self. These smaller goals ranged between “*being around to help raise the children*” (D2; D5), “*getting through the day with few symptoms*” (D4), “*maintaining autonomy*” (D1), and “*keep working*” (D5). Setting short-term goals helped family dyads learning to live “one day at a time”. There was an emphasis on quality of life, rather than its extension. Family members spoke eloquently about their desire to return to simple day-to-day activities that were previously taken for granted.

The family dyads described hope as lively, active, and, above all, a personal choice. The strength of hope was related with positive expectations about the future. Hope was associated with optimistic thinking and a focus on good things to come, but was seen as more than a feel-good emotion. Most of the participants explicitly uttered the words “hope” or “optimism” in their responses. Participants using an optimistic explanatory style interpreted events as external, unstable, and specific. Representative comments about hope and optimism included the following: (D2) “*Being optimistic gives us hope to continue. (…) On the other hand, positive thoughts are necessary for us to deal with everything that is happening to us!*”; (D5) “*There is no point in getting discouraged because things have to move forward (…) things have to move forward*”; (D6) “*We cannot be discouraged because tomorrow things will be better*”.

From the family perspective, hope was the desired possibility, but requires an effort to maintain. At this stage, they perceived a link between faith and hope, a faith present in their daily experience, which made them believe in something positive, based on a transcendent belief. The sense of hope in close connection with the transcendent or the divine was highlighted in the excerpts of some couples: (D4) “*Do not lose faith, have faith and believe that the less good days will be rewarded*”; (D6) “*Believing, believing above all, having faith and believing. Each one goes to seek strength where he wants, one to God and another to the Universe*”.

#### 3.2.2. Barriers

Hope barriers included the limitations imposed by illness, feelings of anguish and helplessness, and poor communication with clinicians.

(a) Limitations imposed by illness

Both patients and carers reported that cancer took over their lives. Participants expressed uncontrolled symptoms, such as pain, constipation, loss of appetite, and weight loss, which jeopardized their hope. While patients experienced cancer symptoms on a daily basis and learned how to accept and adapt to symptoms, carers were frequently distressed by watching patients trying to control their symptoms. Even though some intrusive symptoms reduced their hope, dyads described how “*hope could not be taken away and that it would always be there*” (D4), and that “*despite sometimes it is hard to find her* [hope]” (D1). D5 asserted that “*in spite of the physical discomforts, it’s not worth getting discouraged because things have to move forward*”.

(b) Feelings of anguish and vulnerability

Anguish and vulnerability may lead to feelings of anxiety and fear of death at the EOL, with negative impacts on patient mental health, as they feel a lack of mental and physical control over their lives. D1 shared that, “*Sometimes I feel anguish and sadness, but I don’t want to think too much…*”. D4 added that “*revolt arises in some situations, in an attempt to understand the whys!*”.

(c) Poor communication with clinicians

When clinicians respond to talk of hope from patients by reverting to scientific data about prognosis or survival, patients experience that as a withdrawal of support. D2 believed that a sense of hope could be maintained if the prognosis is coherently communicated: “*The doctor who followed me was a bit negative. A lady of some age. Maybe I have no patience anymore. All that remained was to say ‘look, go order the coffin’, there is nothing to be done*”. Poor communication leads to misunderstandings and can easily damage an often fragile relationship between a patient and health professionals.

#### 3.2.3. Facilitators

Hope facilitators included supportive others, positive thinking and a sense of humour, a connection with nature, faith in religion and science, and a sense of compassion with others and altruism. These are reported more specifically below.

(a) Supportive others

Family, friends, and professional carers fostered hope by making patients feel they were well cared for and supported. Although lack of support was perceived as a barrier to hope, the presence of support from family and friends was frequently cited as helping dyads in their self-management practices and making patients feel valued. Families were also considered part of the participants’ legacy. Family and social support appeared to be a motivator that enabled participants to overcome their ambivalence about seeking palliative care. One dyad said that “*My children are my pillars. If it weren’t for them, I don’t know if I would still be here*” (D1). In addition, D7 stated, “*the whole family is important, especially my brother and younger sister who have always been there*”.

The palliative care team were also viewed as strong sources of support. One dyad said, “*my little angels can get me back on track*” and provide “*positive reinforcement*” (D1). Another dyad mentioned that “*whatever happens, it is in palliative care that we feel supported (…) a person asks and the way they respond gives us security in what they are saying*” (D6). The positive conversations with healthcare providers gave them hope and helped them maintain faith in the treatment process. Hope was fostered by exploring realistic goals with patients, encouraging them to focus on day-to-day living rather than dying, and planning for special times with family.

(b) Positive thinking and sense of humour

Hope was valued as important in regulating negative emotions. Patients suggested that their FCs helped as much as possible, despite the cureless nature and EOL stage of their illness. D2 shared that “*you have to be in a good mood, sometimes life brings more complicated things, and we have to think positively*.” Some patients also comforted their FCs. Preserving a good spirit and humour was essential to them. Some of our participants focused on the optimistic sides of life and everything they could be grateful for. Therefore, through open communication, different elements of hope could be identified in their personality, in order to understand their positive perspective.

(c) Connection with nature

Nature was viewed as a spiritual healer that enables people to reflect on life, making it a valuable element in fulfilling existential needs. D2 mentioned that “*gardening is good because the earth releases energy and that energizes us*”. This notion was echoed in the D7 interview who highlighted the pleasant outdoor space of their home. Some symbols of hope focused on natural elements, such as sounds, smells, and colours. These were highlighted by D7 because “*they reduce pain perception, increase satisfaction, and improve the sense of peace*”.

(d) Trust in religion and medicine

Most participants related their positive expectations to some sort of faith, with a religious belief or a faith in medicine. Hope was considered a divine gift, in the case of D7, who “*trusted God to care for them and take away their suffering*”. Spirituality is a crucial dimension of people’s lives, especially those in the final stages of life. Its importance is recognized as a need of the sick person and family and is considered inherent to palliative care. For D2, “*there is someone who is looking out for us and who gives us support when we need it and who gives us a hand that… There is nothing to fear…*”. D4 stressed that “*prayer was an enormous help*” to them in generating hope.

Other expressions of faith focused on individual doctors, the medical profession, or science: D3 expressed that “*hope does not die if there is faith in medicine (…) God can work miracles through medicine*”.

(e) Sense of compassion with others and altruism

A life of meaning and purpose focuses less on satisfying oneself and more on others. It is a life rich in compassion, altruism, and greater meaning. Patients frequently compare their health condition with that of more severe patients around them and experience positivity with the recovery of other patients. One way in which compassion may boost a patient’s well-being is by increasing a sense of connection to others, especially with family. According to D2, “*sometimes the greatest strength we can receive comes through helping others. Hope is always there for us to draw upon, and it’s easiest to see that when we decide to share it freely with others*”.

## 4. Discussion

This is one of the first studies exploring hope perceptions in patients and carers facing terminal cancer. It highlights important issues for patients and carers individually, but also for the patient and carer as a unit. The major themes and subthemes identified in the interviews suggest that, for family dyads, hope is an inner attribute at the very core of a human being, always present, albeit in varying strengths, depending on factors such as uncontrolled symptoms, feelings of anguish, or poor communication with clinicians. As with other studies of hope in palliative care patients, hope was important and dynamic [40,41,42], working as an element of comfort in palliative care [43].

The findings show that family dyads faced varied threats over the course of their experiences; however, they also identified elements that facilitated hope. Positive interpersonal relationships enhanced hope, by making patients feel they were being well cared for and supported [12,44]. Support from loved ones was fundamental, making patients feel valued and engendering hope [45]. The provision of information from professionals gave them a feeling of security, support, and control, thereby increasing hope [17,46]. A sense of personal worth and meaning in life were also significant. This is consistent with the literature about hope in cancer, confirming the nature of individual attributes of hope, but also the influence professionals have in modulating hopefulness [47].

Considering that hope is a key concept in palliative care, hope-fostering interventions should be developed. A recent meta-analysis [15] revealed that psychosocial interventions increase hope and spirituality and decrease depression in palliative patients. Psychotherapy was the most common type of therapy reported, notably dignity therapy and meaning-centered psychotherapy. Other options included integrative medicine (e.g., art therapy), education, lifestyle changes, and multimodal palliative care intervention (e.g., adjuvant/curative treatment) [15].

In line with Sulmasy et al. [48], for many participants, expressing optimism was deemed important because they believed expressing hope and being optimistic was thought to actually improve the likelihood of therapeutic benefits. As family dyads came to terms with their disease trajectory, hope for themselves was generally transformed into hope for others. Leaving a legacy was often important, ensuring that the dying person’s life was acknowledged as valuable and meaningful and that they had contributed to society [17,49]. Hope in the future of their children continued to be a driving force for many dying patients.

The intersubjective form of hope can be experienced in interpersonal communication, but also in connection with nature. The chance to be close to nature helped patients avoid worrying about their illnesses and provided distractions from negative thoughts and feelings [24]. In a phenomenological–hermeneutic study carried out by Timmermann et al. [50], the authors demonstrated how nature affects a patient’s well-being.

Previous research shows that, when faced with a crisis, people often turn to spirituality or religion as they search for meaning, hope, and love [17]. Feeling useful increased hope in some participants and was seen as closely associated with spirituality [17,51,52]. Other participants understood there is a scientific connection between hope and positive outcomes, even though the actual data are, at best, conflicting [48]. Hope for recovery is not expected in the EOL phase, but rather the acceptance of death, as proposed by Elisabeth Kübler-Ross [53]. This means that patients should recognize the reality of a difficult diagnosis, while no longer protesting or struggling against it [54]. However, patients may withdraw to earlier stages of loss, developed by Kübler-Ross (i.e., denial, anger, bargaining, and depression) or fixate on one of them for some time and feel hope for recovery until they die [1,51]. Even though, accurately, terminally ill patients face an imminent death, family dyads in our study showed a stronger sense of hope. According to Baczewska et al. [51], “it can be assumed then that those who lost hope of recovery take a realistic perspective of their health situation and they probably accept the imminence of death (p. 5). […] Hope of recovery in patients aware of the imminent death is a complex and personal phenomenon, which requires individual strategies of support” (p. 7).

Our participants also expressed a primary hope that they would improve, and a secondary hope that others might also improve. This highlights the importance of research examining the perspectives and experiences of family dyads, including how their responses to suffering might affect their lives and generate a virtuous response to others [55]. These findings align with other studies reporting that altruistic and compassionate individuals use self-effacement to meet the needs of another person [56], often through small, yet impactful acts [55,56,57].

The current study has significant implications for practice. Methods for providing hope-based palliative care support needs urgent consideration. The absence of strategies for providing adequate hope-based care to family dyads with high support needs establishes the need to reinforce and cultivate collaborations between palliative care professionals. A recent study about hope highlighted the relevant role of chaplaincy and spiritual care professionals in witnessing the hope of patients and families [58]. Therefore, supporting hope in dyads implies that palliative care professionals have to bear witness to their own hope, and in particular their own despair, mortality, and powerlessness [58]. Additionally, given the pervasive nature of uncertainty in life among people with advanced illness, witnessing their hope and moving the focus of dialogue away from “living losses”, and towards “living with hope”, has the potential to facilitate a more effective person-centred practice. This practice is grounded on therapeutic communication and involves not only physical issues but also psychological, social, and spiritual needs. Thus, establishing a rapport, building a trusting relationship, concentrating on the salient issues, and applying active listening are vital elements for hope assessment [21,59]. Future research is needed to explore the viability of this approach and evaluate its effectiveness.

All palliative care professionals should receive education, training, and support to improve knowledge and preparation regarding hope-based interventions. In addition, they need to be taught to use humor to foster an emotional atmosphere and help lighten the burden of patients, caregivers, and health care professionals [60]. Lastly, a deeper understanding of hope among dyads, using mixed methods, can inform how hope may alter outcomes in oncological patients and improve comfort in the last stages of life [47]. Moreover, we suggest that realist approaches that draw from critical realism fit the complexity of palliative care practice and help to better understand the nature of nursing work and decision-making [26,28].

### Study Strengths and Limitations

The strength of our study is its methodology, including the rigour of coding, the latent thematic development, and the triangulation of researchers. Within critical realism, this triangulation strategy and intensive cooperation increases credibility and reduces the risk of bias in data interpretation based on pre-conceived understanding and personal opinions [27,28].

Nevertheless, this study has several limitations. First, the findings cannot be generalized and transferred to other populations and contexts beyond the specific group that we interviewed. For example, our sample only included cohabiting heterosexual couples, most of them married. Other important compositions of gay, lesbian, and heterosexual couples engaging in palliative care should be explored in further research.

Second, the recruitment process was challenging, with some potential participants declining to take part due to their frail health. The sample was limited to Catholic participants of European cultural origin and from the central region of Portugal. Different local geography (urban and rural settlements), (in)equity in access to palliative care, and differing legacies of health care allocation can justify different patterns of participant responses. For that reason, palliative patients from different geographic regions and cultural/religious backgrounds, education levels, and incomes may differ in how they describe their hope and how hope is promoted. This is important for more robust interventions in palliative care that measure hope and/or hopelessness as an outcome [15].

Third, the dyads were interviewed once, so there is no information on stability of their attitudes, which were probably significantly influenced by their actual symptom load at the time of interviewing. Furthermore, interviews could have inhibited patients from sharing information they would not want their FC to know. This limitation was addressed by giving patients the freedom to indicate the FC with whom they felt most comfortable.

Although the qualitative method was essential to explore the perceptions of the family dyads, future research should also explore which strategies healthcare professionals can effectively use to foster hope among palliative care patients. Moreover, patients and FCs were asked to recall their experiences of hope during cancer progress until the EOL phase. The possibility of memory and recall bias needs to be considered.

## 5. Conclusions

This study provided insight into how hope is experienced in dyads of EOL patients and their significant others and loved ones. A holistic, person-centred approach to support hope in family dyads experiencing advanced ill health is essential, and must incorporate uncertainty and control as key drivers of the hope process. This care approach will not always be appropriate for everybody, but moving the emphasis of EOL discussion towards the present, with hope, may be a good starting point. Often, health care operates “for” people rather than “with” them, finds it hard to involve people in decisions, and views people’s goals only in terms of clinical outcomes. Therefore, health care professionals should address and maybe even mediate between the patient and carer in their different EOL approaches. Thus, a balance must be struck between respecting the wishes and boundaries of individual patients and FCs, while at the same time having a conversation with those in need, that is not limited to superficial matters.

## Figures and Tables

**Table 1:** Sample description (*n* = 7 family dyads).

Dyad ID Number	Participants (Age in Years)	Living Together	Type of Cancer	ESAS (Mean/SD)
D1	Wife (50, Pt) and Husband (48)	Yes	Metastatic Lung Cancer	4 (1.9)
D2	Husband (57, Pt) and Wife (54)	Yes	Metastatic Colorectal Cancer	3.7 (3.1)
D3	Woman (33, Pt) and Male Partner (37)	Yes	Metastatic Colorectal Cancer	4.9 (2.5)
D4	Wife (54, Pt) and Husband (53)	Yes	Metastatic Melanoma	2.6 (1.6)
D5	Wife (43, Pt) and Husband (46)	Yes	Metastatic Stomach Cancer	2.8 (1.4)
D6	Husband (46, Pt) and Wife (29)	Yes	Metastatic Colorectal Cancer	4.3 (3.6)
D7	Husband (66, Pt) and Wife (65)	Yes	Metastatic Lung Cancer	1.7 (2.9)

Pt—Patient.

## Data Availability

All data generated or analysed during this study are included in this article.
